# Bowel Obstruction Due to the Mesenteric Mass Being an Enteritis Cystica Profunda: A Case Report

**DOI:** 10.7759/cureus.78692

**Published:** 2025-02-07

**Authors:** Meshael S Albahli, Fares Ali M Aljarallah, Ali K Alshaya, Nourah Alabdulaaly, Khaled Altoukhi, Haider A Alshurafa

**Affiliations:** 1 General Surgery, Prince Sultan Military Medical City, Riyadh, SAU; 2 General Surgery, King Khalid University, Riyadh, SAU; 3 Surgical Oncology, Prince Sultan Military Medical City, Riyadh, SAU; 4 Hepatobiliary Surgery, Prince Sultan Military Medical City, Riyadh, SAU

**Keywords:** acute abdomin, enteritis cystica profunda, intestinal obstruction, mesenteric mass, surgical abdominal exploration

## Abstract

Enteritis cystica profunda (ECP) is a rare benign condition characterized by mucin-filled cystic spaces in the submucosal layer of the small intestine. It is often associated with inflammatory conditions, such as Crohn’s disease. This case report describes a 47-year-old male with a history of diabetes and hypertension who presented with flank pain and dysuria. Imaging revealed a mesenteric mass, which led to a differential diagnosis that included malignancies. Despite extensive evaluations, including computed tomography (CT) scans, positron emission tomography (PET) scans, and colonoscopies, the mass showed no significant changes over time. Six months after the initial presentation, the patient returned with acute abdominal pain and signs of bowel obstruction. Surgical intervention revealed a cauliflower-shaped mass at the root of the mesentery, necessitating resection of the small bowel and cecum. Histopathological analysis confirmed ECP. Postoperatively, the patient developed abdominal collections but responded well to treatment, including antibiotics and interventional radiology. This case underscores the diagnostic challenges of ECP, particularly its potential to mimic malignancies, complicating its management in patients with underlying gastrointestinal (GI) diseases. Enhanced awareness and understanding of ECP are crucial for timely diagnosis and appropriate surgical intervention to prevent complications. Continued research is necessary to refine management strategies for this rare condition.

## Introduction

Enteritis cystica profunda (ECP) is a rare benign condition characterized by the formation of mucin-filled cystic spaces within the submucosal layer of the small intestine. This condition is primarily observed in the ileum and jejunum, but it has also been reported in the duodenum [1]. ECP is histologically defined as cystic lesions that are partially lined by nonneoplastic columnar epithelium, distinguishing it from other gastrointestinal (GI) pathologies [2].

The etiology of ECP is not fully understood, but it is thought to arise from the displacement of the glandular epithelium into deeper layers of the intestinal wall, often as a reparative response to mucosal injury or inflammation [3]. ECP has been associated with several underlying GI diseases, including Crohn’s disease and Peutz-Jeghers syndrome, although it can also occur in the absence of any identifiable pathology [4]. This condition was first described in relation to the colonic epithelium, but similar lesions have been documented in various regions of the GI tract, highlighting its broad clinical relevance [2].

ECP is clinically significant because of its potential to mimic more serious conditions, including malignancies. Patients may present with nonspecific GI symptoms such as abdominal pain, vomiting, and bowel obstruction, leading to surgical intervention where histopathological examination is often required for a definitive diagnosis [1].

The prevalence of ECP is notably low, with only a limited number of cases documented in the medical literature. Approximately 15 cases have been reported, indicating its infrequent occurrence [3]. The rarity of ECP, combined with its association with inflammatory bowel diseases, underscores the importance of accurate diagnosis and management, as complications may arise in affected patients [5].

## Case presentation

A 47-year-old male with a history of diabetes mellitus and hypertension and no previous history of surgical procedures presented to the emergency room with flank pain and dysuria, leading to a computed tomography of the kidneys, ureters, and bladder (CT KUB) scan that revealed an incidental mesenteric mass measuring 1.5 × 1.4 cm with a desmoplastic reaction, small bowel thickening, and calcification. Differential diagnoses included carcinoid tumors, sclerosing mesenteritis, and infections.

The patient reported a year-long history of abdominal pain, diarrhea, and episodes of bloody, jelly-like stools, alongside a significant weight loss of 20 kg in one year, and no family history of GI malignancy. Physical examination revealed a palpable mass in the right lower quadrant, and the patient was followed as an outpatient with internal medicine; subsequently, CT of the chest, abdomen, and pelvis (CAP) and CT enterography indicated an unchanged mesenteric mass without metastatic disease.

**Figure 1:**
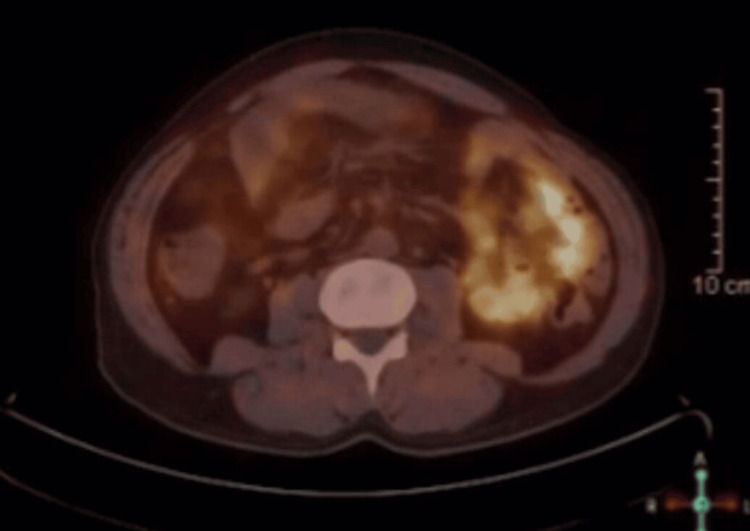
Abdominal positron emission tomography (PET) scan revealed that the small right mesenteric lesion was not Ga-68 DOTATATE avid

The patient was referred to a surgical oncology clinic. The patient continued to experience abdominal pain but tolerated oral intake without obstruction. Further investigations, including tumor markers, positron emission tomography (PET), magnetic resonance imaging (MRI), and colonoscopy, are planned. The PET scan revealed that the small right mesenteric lesion was not Ga-68 DOTATATE avid, making carcinoid lesions less likely (Figure 1). MRI revealed diffuse mural thickening and multiple cystic lesions in the small bowel. The lesion was not accessible for needle biopsy. Two colonoscopies with biopsies from the terminal ileum reported no significant histopathological changes. Enteroscopy revealed a normal small bowel lumen with no intraluminal masses.

After a discussion with a tumor board, a decision was made to repeat the imaging, which revealed no significant changes. Ultimately, the tumor board recommended exploratory surgery due to the high suspicion of malignancy. The patient was informed of the risks associated with surgery and opted for short-term follow-up.

Six months later, the patient returned to the ER with diffuse abdominal pain for 3 days, which started in the umbilical area, then spread throughout the abdomen, and was associated with nausea, vomiting, and watery red diarrhea, accompanied by a 7 kg weight loss in the last three months. Abdominal examination indicated diffuse tenderness and distension. The white blood cell count (6.600 µ/L), hemoglobin (12.7 g/dL), liver profile, and pancreatic enzymes were all normal (Table 1).

**Table 1:** Laboratory results and their normal values

Test	Patient results	Normal values
White blood cells	66,000/mm^3^	5000-10,000/m^3^
Hemoglobin (g/dL)	12.7	13-16
Creatinine (mmol/L)	71	65-119
Blood urea nitrogen (mg/dL)	15	8-24
Albumin (g/dL)	3.5	3.4-5.4
Amylase (U/l)	55	30-110

**Figure 2:**
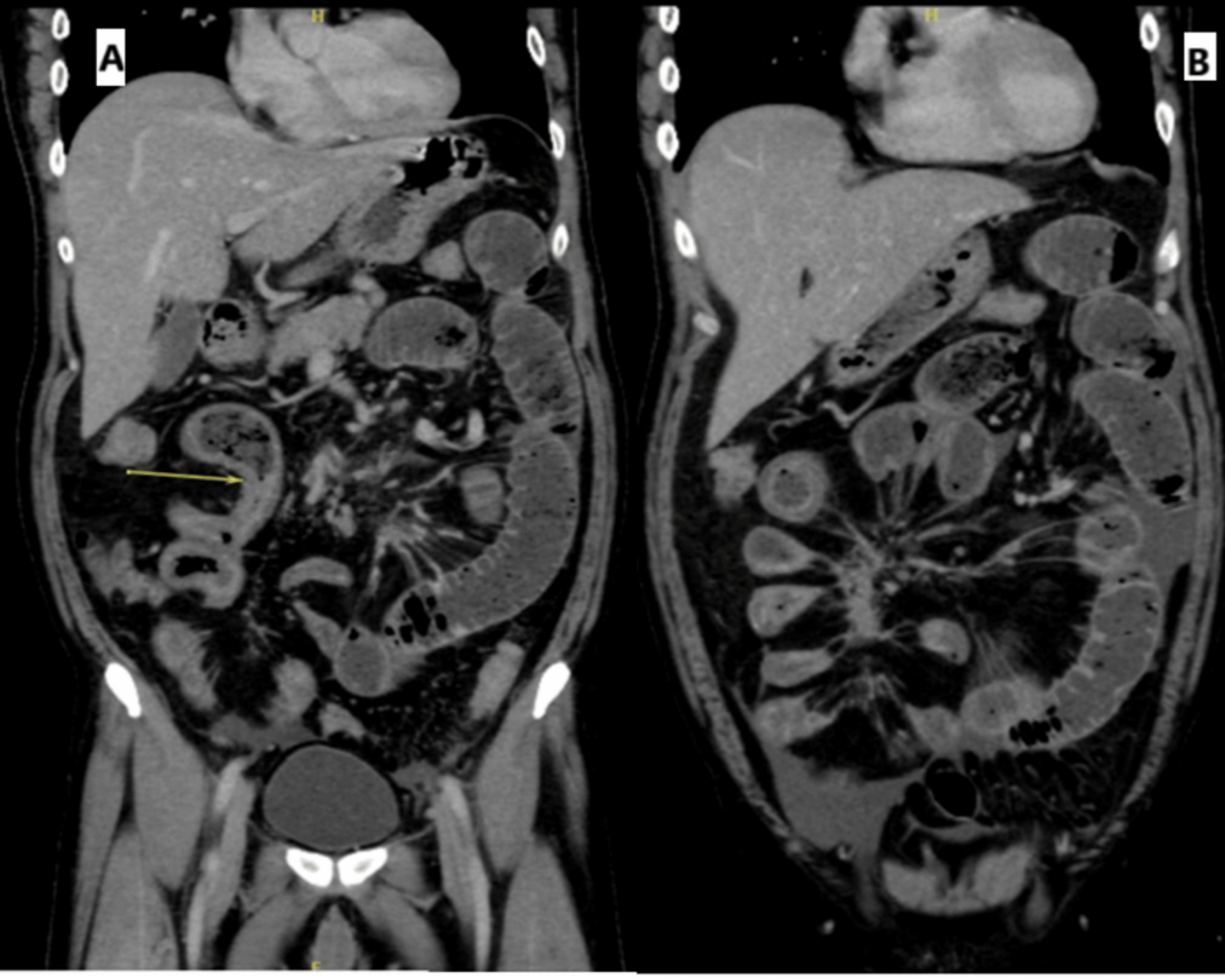
A coronal CT scan showing diffuse dilatation of the small bowel loops measuring up to 4.6 cm within the transition zone It is a long segment of distal/terminal ileal loops that surround and are affected by the desmoplastic reaction and kink from the known mesenteric mass, which shows interval progression of edematous wall thickening that could be reactive to the ongoing obstruction or the mesenteric desmoplastic reaction.

**Figure 3:**
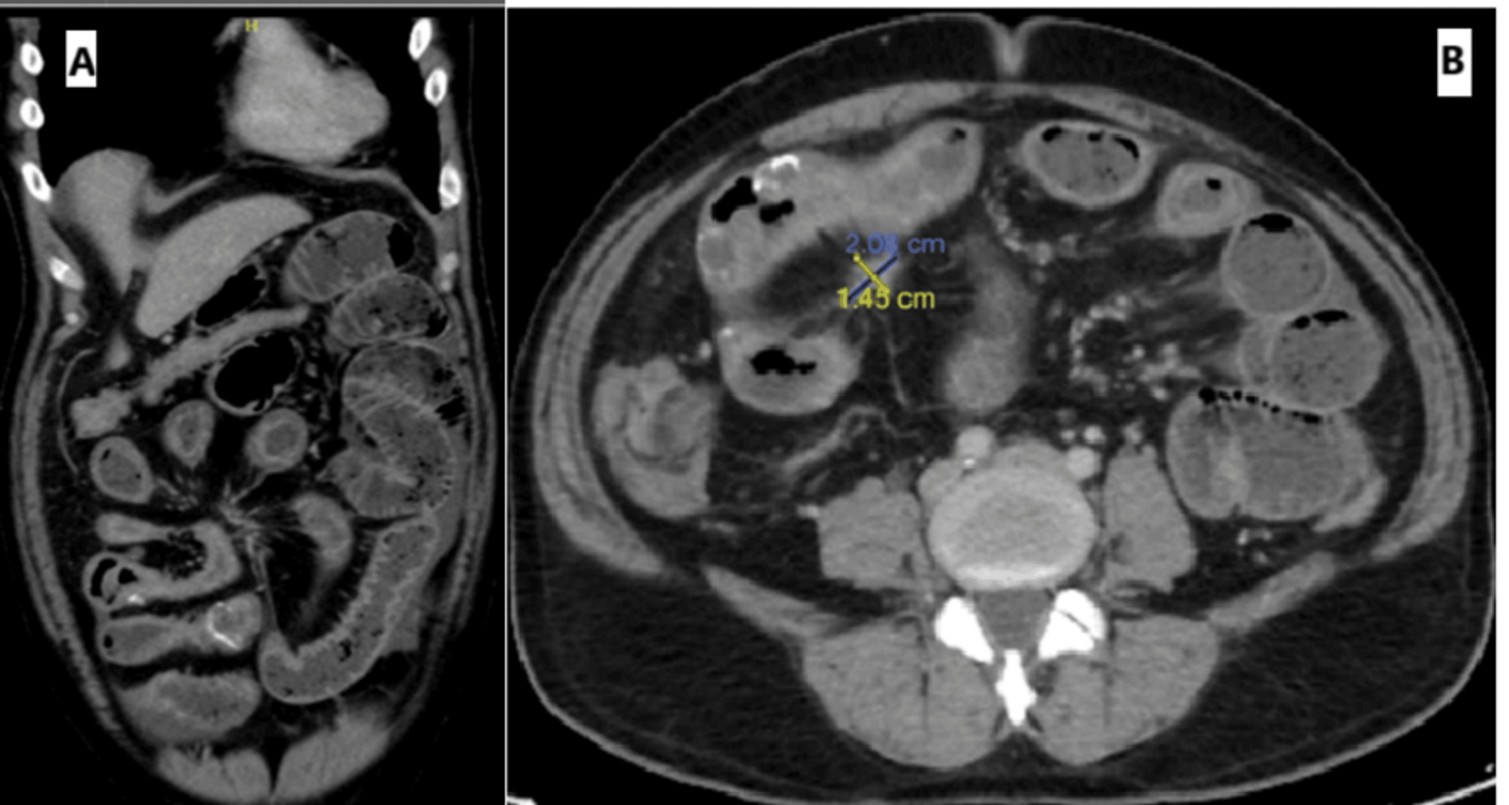
(A) Coronal CT image showing eggshell calcification at the distal ileal loops and (B) axial CT image showing a cauliflower-shaped mass at the root of the mesentery

Contrast-enhanced abdominal tomography revealed diffuse dilation of the small bowel loops, measuring 4.6 cm, with the traction zone beginning at a long segment of the distal/terminal ileal loops, suggesting small bowel obstruction due to the desmoplastic reaction from the known mesenteric mass (Figures 2, 3). The patient was admitted for surgical oncology. Symptom management was initiated with a nasogastric tube. A repeat CT after two days demonstrated progression of the obstruction and edema, with small bowel dilation measuring 5.3 cm in diameter.

**Figure 4:**
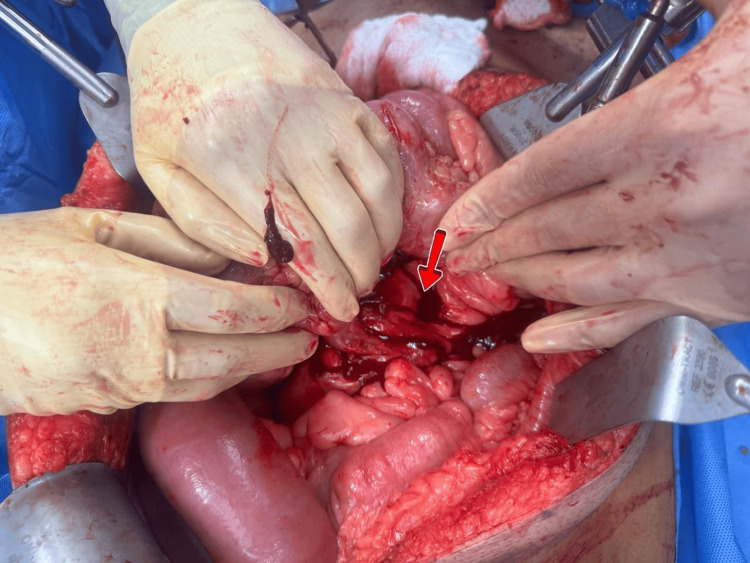
Operative view of the lesion (arrow)

The patient consented for laparotomy in option to resection and anastomosis or ostomy. Exploratory laparotomy revealed a cauliflower-shaped mass at the root of the mesentery of the superior mesenteric artery (SMA), with significant fluid accumulation and small bowel dense adhesions to the mass (Figure 4).

The small bowel and cecum were resected with low ligation of the small bowel 220 cm from the duodenojejunal junction, the mass was taken as one bulk, and vacuum-assisted closure (VAC) was applied for a second look after 24 hours. The follow-up exploration revealed a dilated small bowel with a viable and intact staple site, which was subsequently hand-sewn anastomosed without complications or minimal bleeding.

Postoperatively, the patient experienced no complications, and the obstructive condition resolved. However, persistent abdominal pain and elevated white blood cell count prompted a CT scan one week later, revealing multiple abdominal collections. The patient was treated with IV antibiotics and antifungals and underwent interventional radiology for aspiration of the collections, which was clear and did not indicate intestinal leak. Histopathological analysis during hospitalization revealed ECP. The patient was subsequently discharged in good condition and scheduled for continued follow-up with the oncology surgery service for disease management.

## Discussion

ECP is a rare condition often resulting from inflammatory processes such as Crohn’s disease or trauma. Its association with Crohn’s disease has been documented, highlighting the potential for ECP to mimic malignancies, complicating diagnosis and management [6]. In patients with Crohn’s disease, ECP can present as an obstructive lesion, contributing to the challenges faced in treatment [7]. This association is particularly concerning in cases where the clinical presentation includes abdominal pain and obstructive symptoms, which can lead to misdiagnosis and inappropriate management.

The pathophysiology of ECP involves the displacement of epithelial cells into the submucosal layer following mucosal ulceration or inflammatory damage. This process can create cystic formations that may be mistaken for more serious conditions, including neoplasms [8]. For example, in a reported case, ECP was initially suspected to be a choledochocele, emphasizing the diagnostic difficulty posed by this condition [8].

Furthermore, ECP has been observed in the context of Peutz-Jeghers syndrome, a genetic disorder predisposing individuals to GI polyps and increased cancer risk. The presentation of ECP in such cases may further complicate the clinical picture, necessitating careful monitoring and management strategies to address both benign cystic lesions and the potential for malignant transformation [4].

Notably, ECP can also lead to significant complications, such as intussusception, as documented in pediatric patients [3]. This highlights the importance of recognizing ECP as a potential cause of intestinal obstruction in children and adults alike, which is often overlooked in differential diagnoses.

The management of ECP typically involves a multidisciplinary approach, incorporating surgical intervention when necessary. Surgical exploration may be warranted in cases where there is suspicion of malignancy or if the patient presents with significant obstructive symptoms. The SCARE guidelines emphasize the importance of clear communication regarding surgical risks and the potential for disease progression if left untreated [9].

## Conclusions

In conclusion, ECP represents a unique challenge in GI pathology because of its association with inflammatory bowel disease and its potential to mimic malignancy. This case highlights the complexities of diagnosing and managing ECP. Clinicians must maintain a high index of suspicion for ECP in patients presenting with abdominal pain and obstruction, particularly in the context of Crohn’s disease or hereditary syndromes. Ongoing research and case documentation will be vital in enhancing our understanding of this condition and refining management protocols.
